# Navigating Mental Health: Community Members’ Insights into Social Support and Inclusion in Matsafeni Village in Mbombela, Mpumalanga Province—A Qualitative Study

**DOI:** 10.3390/ijerph21010032

**Published:** 2023-12-25

**Authors:** Nkhensani Eseldah Mboweni, Mabitsela Hezekiel Mphasha, Linda Skaal

**Affiliations:** 1 Department of Public Health, University of Limpopo, Polokwane 0727, South Africa; 200405323@keyaka.ul.ac.za; 2 Department of Human Nutrition and Dietetics, University of Limpopo, Polokwane 0727, South Africa; 3 Department of Public Health, Sefako Makgatho University, GaRankuwa 0204, South Africa; skaal651@gmail.com

**Keywords:** community, social support, community involvement, mental illnesses, family

## Abstract

Mental health illnesses are increasingly prevalent worldwide, and South Africa is no exception. The research investigates the vital significance of social support in enhancing the welfare of individuals dealing with mental disorders. An essential aspect involves comprehending the interplay of emotional and practical supports provided by both families and the larger community. This study aims to explore community perceptions regarding social support and the involvement of individuals diagnosed with mental illnesses in Matsafeni Village. The research employed the qualitative method and descriptive exploratory research design, enabling the researchers to explore social support and the involvement of individuals with mental health disorders. Data were collected through unstructured, open-ended interviews, and participants were selected using a convenience sampling method. A total of only 15 participants were enrolled in this study, with variations in their educational backgrounds. Notably, only two participants had firsthand experiences with family members facing mental disorders. The study underscored the critical roles of family and community supports for individuals diagnosed with mental health disorders. The participants emphasized the necessity of mentally ill individuals receiving support from their loved ones and the broader community. Furthermore, they highlighted the importance for including individuals with mental health disorders in community activities as a means of better managing their conditions. The findings of this study can serve as a foundation for developing interventions to assist and support individuals affected by mental health disorders. Policymakers can also utilize the information to formulate strategies and best practices for promoting mental health awareness within the community.

## 1. Introduction and Background

The rising prevalence of mental health disorders represents a major public health issue with far-reaching social, economic, and human rights implications across diverse global demographics. Mental health disorders, such as anxiety and depression, continue to place a significant burden on individuals and communities, affecting approximately 970 million people worldwide or one in every eight individuals [1,2]. Mental disorders often evoke feelings of fear, confusion, and distress, particularly in their initial stages; and these feelings may be exacerbated by the media’s occasional portrayal of mental health issues in a negative light [3]. For many communities, media and social media serve as the primary sources of information, but these sources often depict mental health conditions negatively, influencing public perceptions, knowledge, and attitudes [4]. It is important to note that although social media can have negative effects, such as cyberbullying and humiliation, it can also serve as a positive force for community building, support, and information sharing when used responsibly and consciously.

The concept of a community, as defined by the World Health Organization (WHO), encompasses a group of individuals who share culture, values, and social norms and reside in the same geographical area [5]. These individuals form a social structure based on connections that have developed over time. The experience of living with a severe mental illness significantly impacts an individual’s preferences for and perceptions of the community. Moreover, community involvement can be viewed not only as a way to distance oneself from experiences and identities associated with illness but also as a process profoundly influenced by the community itself [6].

Despite the growing awareness of mental healthcare in modern society, individuals with mental health disorders continue to face human rights violations, including the use of restraints, denial of privacy, and seclusion. In some countries, these practices are unjustly justified as necessary forms of care [7]. Misconceptions about mental illness can extend to entire families, leading to stigma and the concealment of a family member’s condition [8]. Societal misconceptions and attitudes about mental disorders, as held by an individual’s support network, further complicate mental health conditions. The fear of prejudice and stigma persists in communities owing to the longstanding shame associated with a mental illness [8]. Historically, individuals with mental illnesses have faced stigmatization, ridicule, exclusion, humiliation, and confinement. Despite the increasing awareness, there remain significant barriers to mental health awareness and support [9]. Victims of mental disorders may also endure inhumane living conditions, physical and sexual abuse, neglect, and harmful treatment procedures in healthcare facilities, as documented by the WHO [10]. Consequently, individuals with mental illnesses often live in disadvantaged situations, facing exclusion and marginalization from society, which pose a significant challenge for achieving national and international development goals.

Social and community supports play a pivotal role in maintaining the health and well-being of individuals diagnosed with mental illnesses [11]. This support system often involves a network of relationships with family members, friends, neighbors, and community members, providing emotional, informational, and instrumental assistance. Family members, in particular, often serve as the primary source of support, offering comfort, understanding, and practical help to their loved ones navigating the challenges of mental illness [12]. Social support can manifest in various forms, including emotional support, which involves empathy, love, trust, and caring; informational support, which entails providing advice, guidance, and sharing knowledge to help individuals make informed decisions; and instrumental support, which involves providing tangible resources and services, such as financial aid, transportation, or help with household tasks [13].

Community support encompasses the collective efforts of local organizations, community groups, and neighbors to create an inclusive and supportive environment for individuals with mental illnesses [11]. This can include organizing community events, offering mental health education programs, and establishing peer support groups. Governmental support is equally crucial in providing the necessary assistance and resources for individuals diagnosed with mental illness. This support can take the form of mental health policies, funding for mental health services, and programs aimed at reducing stigma and discrimination. Governments have a responsibility to ensure that mental health services are accessible, affordable, and of high quality, as well as to promote mental health awareness and education [9].

The adoption of mental health policies by governments is driven by the recognition of the substantial societal burden posed by mental illness. Mental health disorders can hinder an individual’s ability to work, study, and participate in social activities, impacting not only their personal lives but also the broader community and economy [9]. Additionally, mental illness often contributes to poverty, as individuals may face financial challenges due to medical expenses, reduced earning capacity, and social exclusion. Moreover, mental illness disproportionately affects the poor, as they may lack access to mental health services, face greater exposure to risk factors, and experience additional barriers to seeking help. Mental health is an intrinsic aspect of overall well-being, and its value is comparable to that of physical health [13]. Therefore, governments have moral and ethical obligations to prioritize mental health as an essential component of public health and human rights. However, government services and policies should not act as barriers to social inclusion but rather as facilitators that empower individuals with mental illnesses to participate fully in their communities. This means implementing policies that address the root causes of mental health disparities, promote social justice, and challenge discriminatory practices.

This study aims to explore community perceptions regarding social support and the involvement of individuals diagnosed with mental illnesses. There is a lack of research specifically focused on community perceptions of social support and the involvement of individuals with mental health disorders in community activities. The use of a qualitative methodology in this research facilitated an in-depth investigation of social support by exploring the experiences and perspectives of community members. Additionally, qualitative methods are adept at capturing the contextual subtleties associated with social support. In contrast, quantitative studies, although beneficial for generalizing findings to broader populations, may oversimplify the intricate nature of social support. They might not grasp the depth, nuances, and contextual elements influencing the quality and efficacy of social support. This study seeks to provide valuable insights into perceptions and the importance for involving individuals with mental health disorders in the local context.

## 2. Research Methodology

### 2.1. Research Method and Design

A qualitative research method was used. Furthermore, an exploratory and descriptive design was used. Fifteen participants, all community members of Matsafeni Village, were individually interviewed to explore their perceptions regarding social support and the involvement of individuals with mental health disorders in community activities. The participants furnished abundant and comprehensive data concerning the involvement of individuals with mental health conditions in community activities and the support they receive.

### 2.2. Study Setting

The study was conducted in Matsafeni Village, located 5 km outside the city of Mbombela in Mpumalanga Province. It has a population of approximately 3723, with 826 households, 51% female residents, and spoken languages including SiSwati (58%), Xitsonga (29%), Sesotho 3%, and Afrikaans 3% [14]. The community has one private clinic, providing limited primary healthcare services. The village was chosen owing to the number of individuals with mental health disorders visibly observed wandering the streets and appearing disoriented and lost.

### 2.3. Sampling and Sampling Size

The target population for this research comprised the inhabitants of Matsafeni Village, specifically those aged 18 and above and actively participating in the community, such as residents, community leaders, and local support organizations. Exclusions were made for individuals facing communication barriers that could impede their ability to convey insights effectively. Utilizing a non-random convenience sampling method, a total of 15 community members from Matsafeni Village were chosen. The sample size was determined by data saturation, which occurred with the 15th participant. At this point, comprehensive and sufficient data had been collected, and no further novel information was forthcoming or anticipated. Consequently, data collection was concluded.

### 2.4. Data Collection Instrument and Procedure

Data were collected using unstructured interviews by employing voice recorders and taking field notes of observed non-verbal cues. One-on-one interviews were conducted using a semi-structured interview guide with open-ended questions to explore the participants’ understanding of mental health and their views on mental health disorders and the causes, symptoms, and available treatments. The questions asked to the participants included, “How do individuals with mental illnesses experience social support within the community?” and “What challenges do individuals with mental illnesses face in participating in community activities?” Probing and clarity-seeking questions were asked to gain a deeper understanding of the responses. Bracketing and reflective remarks were used during the interviews, with the researchers facilitating the interviews, observing non-verbal cues, and taking field notes. The researchers remained neutral, empathetic, and impartial throughout the data collection. In preparation for the actual study, the interviewers were trained in qualitative interview techniques, including open-ended questioning, active listening, and probing, including adherence to ethics. Practical exercises were conducted to hone the interviewers’ skills in eliciting detailed and meaningful responses from the participants. The interviewers were familiarized with the study context to enhance their understanding. Prior to the actual data collection, the interviewers conducted pilot interviews with individuals not included in the study. These sessions allowed for the refinement of interview protocols and techniques. The feedback from the pilot interviews was discussed and incorporated into the final interview approach.

### 2.5. Data Analysis and Measures of Rigor

All the interviews were audio-taped and transcribed. Thematic data analysis [15] was used to identify patterns and themes within the interview data. The researchers and supervisors individually analyzed the transcripts independently before meeting to reach a consensus on the themes and sub-themes. The researchers began by familiarizing themselves with the raw interview data and conducted open coding to generate initial codes, organizing them into potential themes based on similarities. Through continuous review and refinement, the themes were carefully defined, cross-checked for reliability, and visually mapped to illustrate relationships, contributing to a comprehensive understanding of the community’s perceptions of social support and mental health involvement.

Throughout the analysis, the researchers were attentive to the concept of data saturation. This involves assessing whether new data continue to contribute to the identified themes. Once saturation was reached, indicating that no new themes were emerging, the data collection was concluded. The participants’ direct quotations were used to support the findings. Rigor was maintained through trustworthiness, ensuring credibility, transferability, confirmability, and dependability.

### 2.6. Ethical Considerations

This paper forms a part of a larger study on the community members’ perceptions and knowledge regarding mental health disorders in Matsafeni Village, within the Ehlanzeni District municipality in Mpumalanga Province, South Africa. Approval for the larger study was obtained from the Turfloop Research and Ethics Committee (TREC), and a clearance certificate with reference number TREC/624/2022: PG was issued. Permission for the study was also granted by the Matsafeni Traditional Council (Sphezi Royal House). The participants provided signed consent forms confirming their voluntary participation and their right to withdraw at any stage without consequence. The privacy and confidentiality of the participants and their personal information were assured.

## 3. Results

Table 1 depicts the demographic characteristics of the 15 study participants. The age distribution was balanced, with six participants aged 18–35, five aged 36–50, and four aged 50–60. Regarding the participants’ marital status, four were married, four single, two cohabiting, one in a relationship, one never married, and one divorced. Seven participants had completed secondary education, seven had primary education, and one held a tertiary qualification (a degree). In terms of the participants’ employment status, seven were self-employed (vendors—3; shoe repairer—1; construction sector—1; entrepreneurs—2), five were employed (2 part-time; 1 farm worker), and three were unemployed.

Table 2 presents the themes and sub-themes identified from the data. The two main themes were Social Support, with five sub-themes, and Involvement in Community Activities, with two sub-themes.

### 3.1. Theme 1: Social Support

Social support is a critical aspect of the well-being and recovery process for individuals with mental health conditions. It encompasses emotional, practical, and informational assistance provided by caring and understanding individuals. All the participants agreed on the necessity of support for mentally ill individuals. The sub-themes provide detailed insights into the various aspects of support from the community, government, and affected families.

#### 3.1.1. Sub-Theme 1.1: Family Support and Its Importance in Caring for Individuals Diagnosed with Mental Illness

Family and friends play a pivotal role in supporting individuals with mental health conditions by offering understanding, encouragement, and practical aid. Although some participants noted that family support is evident for certain individuals, others observed a lack of support. The following selected participant quotes illustrate these differing views:

**50–60-year-old, unmarried woman**: “*Families can support their loved ones with mental illnesses by ensuring they receive medical care, adhere to treatment routines, and maintain personal hygiene.*”;
**18–35-year-old married man**: “*Some people don’t seem to be cared for by their families, as they are often untidy and foraging for food. This lack of family support hinders community involvement.*”;
**36–50-year-old cohabiting man**: “*I haven’t seen community members supporting people with mental illnesses, which may stem from a lack of familial care and love.*”;
**36–50-year-old cohabiting woman**: “*While some are well cared for by their families, others cannot be managed and become violent. Those who wander make it challenging for families to provide consistent care and support.*”.

#### 3.1.2. Sub-Theme 1.2: Community Support and Its Importance to Individuals Diagnosed with Mental Disorders

Community support is vital for the well-being of individuals with a mental illness and their families. It includes resources and advocacy from neighborhood associations and regional programs. The participants emphasized the need for community members to assist those with mental health disorders in managing their condition. Some members of the Matsafeni community actively support mentally ill individuals and their families, as evidenced by the following quotes:

**50–60-year-old, unmarried woman**: “*For those lacking family support, the community should facilitate hospital admissions and provide food to prevent scavenging from garbage bins.*”
**50–60-year-old divorced woman**: “*People’s attitudes vary; some empathize with the mentally ill and offer assistance, while others find amusement in their plight, leading to mistreatment and isolation.*”;
**18–35-year-old married man**: “*Community members can support affected families by guiding lost individuals home and showing care without discrimination.*”;
**18–35-year-old married woman**: “*I know individuals with mental health disorders who have received support in the form of food and clothing from neighbors and community members.*”;
**36–50-year-old cohabiting woman**: “*The community can assist by providing food and clothing to those living on the streets.*”.

#### 3.1.3. Sub-Theme 1.3: Family’s Inability to Support Their Family Member with a Mental Disorder and Its Impact on Community Support

The participants expressed concerns about the difficulty for intervening and assisting when some families prefer not to involve the community in their matters. The following quotes highlight these concerns:

**36–50-year-old woman in a relationship**: “*Educating the community about mental disorders is crucial, but it can be challenging to assist families who prefer not to accept help or intervention from others.*”;
**50–60-year-old divorced woman**: “*The community should look out for each other and report the whereabouts of mentally ill individuals, but assistance is often dependent on the family’s willingness to accept help.*”.

#### 3.1.4. Sub-Theme 1.4: Attitudes of Community Members toward Supporting Individuals with Mental Disorders and Their Families

The study suggests that some community members perceive mental illnesses as personal problems and prefer not to get involved. The following quote from Participant No. 10 illustrates this view:

**18–35-year-old single man**: “*If a problem is not yours, stay out of it. Getting involved in your neighbors’ or community’s issues can lead to your own mental health problems.*”.

#### 3.1.5. Sub-Theme 1.5: Governmental Support to Individuals Diagnosed with Mental Disorders

Governmental support is essential for ensuring access to quality care, reducing stigma, and promoting overall well-being for individuals with mental health conditions and their families. Although the participants acknowledged government programs to support mentally ill individuals, there was a lack of awareness about the specific programs. The following quotes reflect these sentiments:

**36–50-year-old woman in a relationship**: “*The government should fund and provide necessary treatment services to help mental health patients recover.*”;
**50–60-year-old married man**: “*The government should assist with medication and treatment for mental health patients.*”;
**18–35-year-old married woman**: “*Although I think there should be other governmental support programs, I’m not aware of any.*”.

### 3.2. Theme 2: Involvement in Community Activities

Engaging individuals with a mental illness in community activities is crucial for their well-being, social integration, and empowerment. Most participants believe that people with mental health conditions should participate in community activities. However, some expressed opposing views, highlighting social rejection and stigmatization. The following sub-themes below provide insights into these perspectives:

#### 3.2.1. Sub-Theme 2.1: Participation of Mentally Ill Individuals in Community Activities

The majority of the participants felt that people with mental illnesses could be involved in community activities, although the extent of participation may depend on the severity of the disorder or the individual’s level of functioning. The following quotes represent various viewpoints:

**50–60-year-old married man**: “*People with mental illnesses should not be excluded from community activities. Inclusion can encourage those with substance-abuse-related illnesses to feel valued.*”;
**36–50-year-old married man**: “*They should be involved in social activities, but care must be taken as some activities might be overwhelming. Keeping them engaged can help redirect their focus and have a positive impact.*”;
**18–35-year-old single woman**: “*Not all mentally ill people can be involved in community activities, as some may not have any interest. Some may refuse to stay at home or seek help, making involvement challenging.*”;
**50–60-year-old divorced woman**: “*Mentally ill individuals, especially those not severely affected, can participate in community activities. However, their contributions may be limited.*”;
**18–35-year-old married man**: “I*t depends on the type of illness. Some may find it impossible to participate, but those who can actively engage should be given the opportunity.*”;
**50–60-year-old married man**: “*My sons, who have mental health conditions, are involved in community activities, like church, and they are not excluded or isolated.*”;
**36–50-year-old cohabiting man**: “*People with mental illnesses should be involved in community activities, such as attending church services, which can aid in their recovery.*”.

#### 3.2.2. Sub-Theme 2.2: Social Rejection and Stigmatization

Some participants expressed concerns about involving mentally ill individuals in community activities owing to potential disruptions and the prevailing stigma. The following quotes illustrate these concerns:

**36–50-year-old woman in a relationship**: “*Involving mentally ill individuals, especially those with minor disorders, may be difficult as they may misunderstand the invitation. Those with major mental disorders may be incapable of participating.*”;
**50–60-year-old, unmarried woman**: “*Mentally ill people should not be involved in community activities, as they may not be able to participate effectively and could cause disruptions.*”;
**50–60-year-old married man**: “*I don’t think people with mental disorders should be involved in any community activities. I prefer to avoid them.*”;
**36–50-year-old cohabiting woman**: “*People with mental health disorders may not comprehend the proceedings of community activities. While they may invite themselves, they could cause disruptions.*”.

## 4. Discussion

The study found that the Matsafeni community recognizes the critical importance for providing support to individuals with mental illnesses and their families as being essential for recovery. A robust support network, comprising individuals whom one likes, respects, and trusts, is a crucial component of mental rehabilitation. Moreover, it is vital for individuals to have someone with whom they can comfortably share their experiences and seek help. This support could come from various sources, such as family members, friends, teachers, church leaders, neighbors, or classmates [16]. The findings suggest that families typically serve as primary caregivers and do provide care and support for their loved ones diagnosed with mental illnesses. In fact, one in every four households has at least one person suffering from a mental illness, with families often playing the role of the primary caregivers [17]. However, some participants noted that individuals with mental illnesses, and found wandering the streets, often lack familial support through their illnesses.

Moreover, the study underscored the importance of practical support for people with mental health disorders, which includes providing tangible aid. This may involve assisting with daily tasks, transportation to appointments, medication management, or household chores. Given that individuals suffering from depression, anxiety, and substance-abuse disorders may struggle with compliance and adherence to treatment, such support becomes imperative. Individuals who receive practical support find it easier to navigate daily stressors and follow their treatment plans. The attention and support from friends and family significantly enhance the treatment’s effectiveness and greatly improve recovery chances [18]. Furthermore, the study highlights the need to establish support systems that encourage medication adherence and course completion and create minor employment opportunities to enable individuals to sustain themselves. Additionally, the study highlights the need to conduct longitudinal studies to track the outcomes of individuals who have received support through the proposed systems over an extended period and investigate the sustainability of improvements in medication adherence, course completion, and economic empowerment, providing valuable insights into the long-term impacts of support systems. A mixed-method research approach is warranted to evaluate the effectiveness of the intervention and explore the participants’ individual experiences, the encountered obstacles, and the perceived influence on their lives. Despite indications that the community provides assistance to individuals grappling with mental disorders, and their families, the findings of the study highlight the potential for further improvements and enhancements in this support system. Hence, it is essential to routinely gather input from community members to pinpoint areas for enhancement and adjust strategies accordingly. Furthermore, consistent mental health awareness campaigns within the community are crucial for enhancing and refining the support that is provided. Therefore, increasing knowledge and awareness of mental health issues is crucial for the community as a whole.

Educational programs, workshops, and public awareness campaigns can help dispel myths, reduce stigma, and raise awareness of mental health issues and the available support resources. In addition, actions to enhance the policy environment and strategic communication for network development, stakeholder engagement, mental-health-literacy improvement, and behavioral change are all integral aspects of mental health promotion [19]. Emotional support for individuals with mental health issues involves empathy, understanding, and reassurance. It requires active listening, providing comfort, and respecting their feelings and circumstances. Because everyone’s emotional experience is unique, it is important to embrace and appreciate each individual. It has been noted that providing emotional care to the mentally ill requires delicacy, encouraging them to share their feelings, and listening without judgment [20]. People who receive emotional support during their struggles feel heard, valued, and less alone. Being heard helps mental health patients engage more effectively in their treatment [21].

However, the study also identified limitations to the provision of support. Some participants highlighted challenges from the families of affected individuals, who might deny the community access to their affairs, and from the affected individuals themselves, who might refuse help or discontinue treatment, especially those affected by substance-abuse-related mental illnesses. It should be noted that if society fails to recognize the burden of mental disorders on affected families, support for them may be limited [22]. The study also revealed that some families or parents still conceal their mentally ill loved ones, often to shield the family from shame and embarrassment [23]. Moreover, social and cultural changes have led to a diffusion of communal responsibility, where community values and norms are overlooked and individuals within the community may not concern themselves with others’ problems. The study also found that people’s perceptions of mental health disorders influence their attitudes toward sufferers of mental illnesses. Communities have various understandings of mental health disorders, often informed by negative perceptions that lead to the labeling and stigmatization of people with mental health disorders [24].

Governments can create comprehensive mental health laws and policies that prioritize mental health as a public health issue. These regulations should ensure the integration of mental health into general healthcare systems, protect the rights of individuals with mental illnesses, and promote equitable access to mental health treatments. When aligned with human rights principles, mental health legislation provides a legal framework to address critical mental health issues, such as access to care, rehabilitation, the integration of individuals with mental disorders into society, and the promotion of mental health across various societal sectors [25]. The participants indicated that the government should fund and provide the necessary treatment and that some support systems from the government are unknown to the public. This signals the need for public awareness campaigns on mental health services. Many individuals in South Africa who suffer from mental health illnesses are either unaware that effective treatments are available or may not have access to the necessary care provided by the government [26]. The participants recommended that the government should offer family support programs designed specifically to help families overcome challenges and build resilience. These programs could include counseling, psychoeducation, respite care, and support groups.

Overall, the participants had a positive attitude toward individuals with mental illnesses participating in community activities, such as church services, soccer matches, and community meetings. As social beings, individuals are not meant to live in isolation [27]. Communities are crucial for individuals to thrive, particularly for those with mental illnesses and who often experience feelings of loneliness and isolation. The inclusion of people with mental illnesses in communities involves encouraging them to participate as independently as possible in activities they enjoy and providing them with valued ways to contribute [28]. Although individuals with a mental illness may face stigma or discrimination that limits their community participation, involving them in activities, such as work, volunteering, social groups, or leisure activities, can enhance their quality of life and foster recovery for those with a severe mental illness [29].

## 5. Conclusions and Recommendations

This study emphasized the pivotal role of family support in the well-being of individuals diagnosed with mental disorders. It delved into the crucial contributions of emotional and practical support from families, community assistance, and government initiatives in aiding these individuals. The research highlighted that a lack of sufficient family support could result in limited community assistance, detailing diverse attitudes within the community toward supporting those with mental disorders. Additionally, the study indicated the obstacles faced by individuals with mental illnesses in engaging in community activities owing to social rejection and stigmatization, coupled with a lack of awareness about government programs dedicated to mental health support.

In light of these findings, the study put forth recommendations for increased investment in mental health services, the implementation of community-based mental health education programs, the establishment of effective and well-equipped and funded support systems, and the initiation of public education and anti-stigma campaigns. The research stressed the significance of a comprehensive approach, encompassing awareness, education, robust support systems, and policy changes, to foster a supportive and inclusive environment for individuals grappling with mental health conditions.

## 6. Limitations

The study relied on self-reported data, which may be subject to biases and social desirability effects. It would be beneficial for this study to be replicated in other areas of the province and to be conducted at the provincial level for broader findings on the subject matter.

Despite these limitations, the study provides valuable qualitative insights and can serve as a basis for further research and interventions.

## Figures and Tables

**Table 1:** Demographic background.

Participant Identifier	Age Group	Gender	Marital Status	Level of Education	Firsthand Experience	Employment Status
Participant 1	50–60	Male	Married	Grade 11	No family with condition	Self-employed—Shoe repairer
Participant 2	18–35	Male	Single	Grade 10	No family with condition	Employed—Shisanyama
Participant 3	36–50	Male	Married	Grade 2	No family with condition	Self-employed—Construction
Participant 4	36–50	Female	In a relationship	Grade 6	Family with condition	Self-employed—Vendor
Participant 5	18–35	Female	Single	Grade 8	No family with condition	Street Vendor
Participant 6	50–60	Female	Never married	Grade 5	No family with condition	Employed
Participant 7	50–60	Female	Divorced	Grade 4	No family with condition	Street Vendor
Participant 8	36–50	Female	Married	Grade 8	No family with condition	Entrepreneur—Glass cutting and fitting
Participant 9	18–35	Male	Married	Degree	No family with condition	Entrepreneur—Bugler and aluminum
Participant 10	18–35	Male	Single	Grade 12	No family with condition	Unemployed
Participant 11	50–60	Male	Married	Grade 5	Family member with condition	Farm Worker
Participant 12	18–35	Female	Married	Grade 8	No family with condition	Unemployed
Participant 13	36–50	Male	Cohabiting	Grade 4	No family with condition	Part-time employment
Participant 14	36–50	Female	Cohabiting	Grade 4	No family with condition	Part-time employment
Participant 15	18–35	Male	Single	Grade 12	No family with condition	Unemployed

**Table 2:** Themes and sub-themes that emerged from the data.

Themes		Sub-Themes
Theme 1	Social Support	3.1 Family support and its importance to individuals diagnosed with mental disorders
		3.2 Community support and its importance to individuals diagnosed with mental disorders
		3.3 Family’s inability to support their family member with a mental disorder and its impact on community support
		3.4 Attitudes of community members toward supporting individuals with mental disorders and their families
		3.5 Governmental support to patients living with mental disorders
Theme 2	Involvement in Community Activities	4.1 Participation of mentally ill individuals in community activities
		4.2 Social rejection and stigmatization

## Data Availability

The data collected for this study are held securely and are accessible only to the research team. The findings of this study emanated from data collected from the participants sampled in Matsafeni Village, Mbombela, Mpumalanga Province. Thus, the data that support the findings of this study are not publicly accessible.
